# MyPlate, Half-Plate, and No Plate: How Visual Plate-Related Dietary Benchmarks Influence What Food People Serve

**DOI:** 10.7759/cureus.25231

**Published:** 2022-05-23

**Authors:** Brian Wansink, Audrey Wansink

**Affiliations:** 1 Behavioral Psychology, Cornell University, Ithaca, USA; 2 New Visions Program, Lansing High School, Lansing, USA

**Keywords:** federal nutrition programs, treating obesity, family meals, behavioral interventions, half-plate, myplate, nutrition education, weight loss and obesity, eating behaviors, dietary guidelines

## Abstract

Introduction

The objective of this article is to analyze whether visual plate-related dietary guidance systems - such as the MyPlate guideline or the Half-Plate Rule - help people eat better when dining at home or in restaurants.

Methods

To help explore this, 104 young adults were randomly assigned to follow either (1) USDA MyPlate guidelines, (2) the Half-Plate Rule, or (3) no guidelines (control condition). They then used their assigned guidelines to complete the survey while eating a dinner of their choice. They completed a food diary for the meal and then completed a survey about their experience.

Results

Both the two visual dietary guidance systems (My Plate and the Half-Plate Rule) were considered easy to understand and easy to follow, and they left people with fewer questions about what to eat (all p < 0.01). Understandability is important because those people who rated a system "easy to follow" indicated they had consumed less meat than usual (r = 0.268), but understandability was uncorrelated with fruit and vegetable intake (r = 0.092) and carbohydrate intake (r = 0.069).

Conclusions

There are three key conclusions to these and other findings: first, the simplest guidance system may be more effective than none. Second, even the most perfect dietary guidance system will not change behavior if (a) the foods are not available, or (b) it is not followed. Third, guidance systems could *over-*increase the consumption of some foods (such as dairy) they specifically mention, presumably because it makes them more salient in one's mind.

## Introduction

Eating balanced, nutritious meals at home is difficult for all ages - from young people who are just leaving home as well as elderly people who are increasingly trying to stay at home as they age. If used strategically, technology platforms that are built on behavioral science principles and testing can help people eat healthier [1,2]. Recent trends in behavioral science and nutrition research have focused on how the "rules of thumb" might prove to be useful in helping individuals make better meal-related decisions, whether at home or dining out [3]. Specifically, how do different simple visual dietary guidance systems such as the MyPlate or the Half-Plate Rule influence eating behaviors compared to people who follow no eating guidelines?

One useful way to address mindless eating is to provide a dietary guidance system to help people quickly determine which foods to eat at the appropriate time [3]. Although dietary guidance is largely available through websites and apps, people need nutrition guidelines or rules of thumb that can be quickly remembered and used at the moment [4]. One example of such dietary guidance is the U.S. Dietary Guidelines. Until 2009, this guidance system was graphically represented by an image of a food pyramid, which was referred to as "MyPyramid." In 2009, this pyramid was modified into the form of a plate that was proportionally divided into four quarters that represented components of grains, proteins, fruits, and vegetables (along with a serving of dairy on the side of the plate, which was represented as a glass of milk). This system became adopted most quickly by those who had more years of college, had children, or believed that this guideline would work for their health [5]. It is important to note that people in this study were self-reporting their adoption of the system, and there was no indication of how extensive or persistent their adoption of it was.

Since its introduction, the MyPlate icon has been widely used by the US to represent the more lengthy and complete 149-page dietary guidelines. Considering that nutrition information sometimes seems too complex to be actionable [6,7], MyPlate was designed to offer a quick visual and actionable summary and an easy-to-follow benchmark. In doing so, it was intended to prompt diners to think about eating more balanced meals, such as ones that included more fruits, vegetables, and whole grains [8]. Yet there is a concern that some people may not be following any dietary guidance system [2]. For example, if they lack motivation and nutrition knowledge, they may not understand how to categorize their food into the recommended components: grain, protein, fruits and vegetables, and dairy [5]. Moreover, even when users of MyPlate or MyPyramid do understand a rule, issues like individual food preferences and tastes could influence whether they actually follow these guidelines. Therefore, it is necessary to examine not only whether people understand the guidelines but also whether they follow them. For instance, a person might understand that they should eat more vegetables, but if there are no vegetables in one's refrigerator or pantry, they cannot translate this understanding into consumption.

A second rule-of-thumb dietary guidance system - the Half-Plate Rule - is complementary to the MyPlate approach [9]. It is a more basic system that has been utilized in school cafeterias, dining halls, and grocery stores (Wegman’s grocery stores adapted the Half-Plate Rule, renaming it Half-Plate "Healthy"). The Half-Plate Rule recommends that whenever a person makes an eating decision (such as what to order or what to serve themselves), they should aim for half of their plate to be filled with vegetables, fruits, or salad, while the other half should contain a reasonable balance of anything else [9]. The key question is: will such guidance systems provide people with the confidence to eat better and lead them to consume less food and have more relatively balanced nutrition meals?

When interventions and instructional advice are given, it is often believed that the simpler the approach, the higher the adherence [9]. For instance, simplicity and unambiguousness could be two reasons why simple or single-food diets (such as the soup diet or grapefruit diet) achieve quick success with some people. That is, by overly explicitly raising one’s awareness of the variety of foods, might people eat more of those foods than they otherwise would. Categorization research has shown that the more categories are presented to people, the more foods they take, and this explains some findings suggested in behavioral economics [10]. One concern with applying this bias to a food selection guidance system would be if a dietary system could lead people to overeat some food items by specifically pointing them out and bringing them to the front of one's awareness. This would not be a problem with foods that are typically not overeaten, such as fruits and vegetables. It would be a problem, however, for foods that are easy to overeat, such as meat, grains, and dairy. A system that makes these highly salient might also make them highly consumed.

This research aims at determining how dietary guidance systems might influence diners to consciously eat better. In doing so, this offers an important way that Smart Homes, apps, and technological platforms could also communicate or track eating behavior in a helpful way. In doing so, the results of this study can also give health professionals and public health officials insights into the types of guidance that they can use to more effectively influence eating behaviors. Such findings would also contribute to the meaningful debate on whether it is more effective to think about how to best present dietary guidance information.

This article was previously posted to the medRxiv.org, researchgate.net, and SSRN.com preprint servers in July-August 2021.

## Materials and methods

To initially determine the effectiveness of dietary guidance systems, 104 university students and staff were offered extra course credit if they agreed to be involved in an eating study during a four-day holiday break. In this IRB-approved study, these individuals were randomly divided into one of three conditions. One group of participants was asked to follow the MyPlate guide system recommended by the USDA. Another group was asked to follow the Half-Plate rule. The third group was asked to eat as they normally would (control condition). These instructions were briefly summarized on a single page of the paper, which also included a graphic icon of the dietary guidance system (a MyPlate divided into four or a MyPlate divided into two).

Those people in the MyPlate guidelines condition were presented with the MyPlate icon, and they were advised to have balanced amounts of fruits, vegetables, grains, proteins, and dairy for lunches and dinners. The MyPlate guidelines have been adopted by the US government since 2009. Those people in the Half-Plate rule condition were shown a graphic of the Half-Plate, and they were advised to fill half of their dinner plates filled with fruits, vegetables, and salad, and the other half can include foods they wish. They were also told consumption amount was not an issue and they could freely refill their plates, but then still needed to follow this Half-Plate rule. Those people in the third group were in the control condition, and they were given no guidance or rules as to what to eat.

After finishing their meal under these randomly assigned conditions, participants answered a one-page survey questionnaire that had been sent home with them. Questions were asked using a 9-point scale (1 = Strongly Disagree; 9 = Strongly Agree). The first three questions asked participants to rate how easy it was for them to understand and follow the assigned rule, and whether they had any questions about it. After that, participants were asked to rate questions regarding their eating behaviors, such as "I ate healthier than usual," "I ate less food than usual," "I ate more fruits and vegetables," "I ate less dairy than usual," and so on. The final part of the survey required participants to estimate the total calories that they ate and to answer how many different foods they ingested, as well as the snacking calories. They were told to use this rule at any or all meals they consumed during that four-day period, but they were to answer the survey immediately after one specific evening dinner meal that was not a special holiday meal or celebration meal. They could select whichever dinner they wished.

Among those participants who initially agreed to be involved in the study, 69 analyzed an at-home meal, and the other 35 individuals analyzed meals at restaurants. The foods that respondents had reported eating were categorized for analysis, but because many people did not include specific enough indications of their serving size, these data were not ultimately analyzed. Similarly, although self-reported measures of calorie intake were asked in the questionnaire, such measures can be highly variable and inaccurate. Although one way to analyze these data is to exclude outliers, it was believed to be more prudent to include summary measures of them in the tables but not to emphasize them in the discussion of the analyses.

The analyses were conducted using the Wizard (Version 1.9.48) Statistical Analysis software to assess the mean value of rating scores and self-reported calorie intakes. We performed independent t-tests to test the difference between the individual conditions. The Pearson correlation test was also conducted to examine if there was any relationship among the variable variances.

## Results

As shown in Table 1, when comparing participants who used a guidance system such as the Half-Plate or MyPlate guidelines versus those following no dietary guidance system (control condition), it is found that using dietary guideline systems generally led participants to have fewer questions about what to eat. Of the two dietary guidance systems, those using the Half-Plate rule reported that it was easier to follow (6.59 vs. 5.12; t (45) =2.37, p=0.022) and easier to understand (8.62 vs. 7.68; t (45) =2.02, p=0.049) than the MyPlate guidelines.

**Table 1:** How dietary guidance systems influence at-home eating behaviors ^1^Standard deviations in parentheses; *p<0.05, **p<0.01; rating scale: 1 = strongly disagree to 9 = strongly agree; cell sizes vary by the question since not all questions were answered by all respondents

	Half-Plate Rule n=35	MyPlate n=35	Control n=34	F-test (2,103)	P-value
It was easy to follow this rule^1^	5.41 (0.74)	5.03 (0.69)	6.81(0.65)	7.61	0.001**
It was easy to understand this rule^1^	6.85 (0.75)	7.67 (0.50)	8.31 (0.26)	3.047	0.001**
I had questions about it^1^	4.50 (0.89)	4.03 (0.80)	2.64 (0.65)	6.31	0.003**
I ate healthier than usual^1^	5.53 (0.68)	5.58 (0.56)	5.83 (0.77)	0.024	0.890
I ate less unhealthily than usual^1^	5.44 (0.67)	5.0 (0.68)	4.61 (0.76)	1.48	0.249
I ate more fruits, vegetables, and salad^1^	6.12 (0.61)	6.00 (0.73)	6.25 (0.77)	3.085	0.878
I ate fewer carbs than usual^1^	4.79 (0.64)	4.75 (0.71)	5.50 (0.82)	1.383	0.256
I ate less meat than usual^1^	4.71 (0.69)	4.25 (0.68)	5.20 (0.79)	1.785	0.173
I ate less dairy than usual^1^	4.65 (0.77)	3.60 (0.76)	4.90 (0.84)	3.722	0.030*
I ate fewer desserts than usual^1^	5.61 (0.81)	5.53 (0.97)	6.33 (0.85)	1.05	0.357
I snacked less than usual^1^	5.59 (0.71)	5.56 (0.65)	4.69 (0.69)	2.284	0.107

People who believed that their dietary guidance system was easy to follow tended to report eating slightly better. As Table 2 indicates, there was a significant correlation between a system being easy to follow and a person eating less meat than usual during this consumption period (r=0.268; p<0.05). In contrast, having an easy-to-follow system had no impact on whether a person reported eating healthier eating (r=0.034; p>0.05), eating more fruits and vegetables (r=.092; p>.05), or eating fewer carbohydrates (r=0.069; p>0.05).

**Table 2:** Pearson’s correlations between eating behaviors* *Correlations above r=0.20 (p<0.05) and r=0.26 (p<0.01). ^1^Rating scale: 1 = strongly disagree to 9 = strongly agree; cell sizes vary by question since not all questions were asked by all respondents.

	Easy follow	Easy understand	Had questions	Healthier usual	Less unhealthy	Snacked less	More fruit/veg	Less carb	Less meat	Less dairy	Fewer desserts	
												Easy to follow^1^	1.000	0.217	−0.266	0.034	−0.108	−0.139	0.092	0.069	0.268	0.091	−0.006	
Easy to understand^1^	0.217	1.000	−0.509	0.041	−0.170	−0.036	−0.056	0.025	−0.107	0.000	−0.068	
Had questions^1^	−0.266	−0.509	1.000	0.075	0.289	0.171	0.140	0.116	0.182	0.069	−0.045	
Healthier than usual^1^	0.034	0.041	0.075	1.000	0.561	0.447	0.607	0.537	0.505	0.252	0.462	
Less unhealthy^1^	−0.108	−0.170	0.289	0.561	1.000	0.463	0.400	0.404	0.455	0.210	0.273	
Snacked Less^1^	-0.139	−0.036	0.171	0.447	0.463	1.000	0.386	0.316	0.185	0.165	0.287	
More fruit and vegs^1^	0.092	−0.056	0.140	0.607	0.400	0.386	1.000	0.453	0.433	0.141	0.384	
Less carbs^1^	0.069	0.025	0.116	0.537	0.404	0.316	0.453	1.000	0.458	0.220	0.311	
Less meat^1^	0.268	−0.107	0.182	0.505	0.455	0.185	0.433	0.458	1.000	0.357	0.229	
Less dairy^1^	0.091	0.000	0.069	0.252	0.210	0.165	0.141	0.220	0.357	1.000	0.562	
Fewer desserts^1^	−0.006	−0.068	−0.045	0.462	0.273	0.287	0.384	0.311	0.229	0.562	1.000	

Yet even though there were few positive correlations between being easy to follow and eating better, neither of the two individual guidance systems translated into people reporting they ate better. People who followed either of the two dietary guidance systems did not report that they ate any healthier (p>0.20) than those following neither system. As Table 1 indicates, the one exception was that those people who were given MyPlate guidelines ate the most dairy when compared to usual (3.60 vs. 4.65 and 4.90; F (2,103) = 3.722, p=0.03).

Following the study, debriefing interviews were conducted. They revealed an unexpected explanation as to why dietary guidance systems improved understanding of how to eat better but then had little impact on actual eating behavior. These interviews indicated that many of these meals did not have a wide variety of fruits and vegetables available. As a result, there was no opportunity to substitute them for carbohydrates, grains, or meat and protein in the way suggested by the guidelines. This made following either the MyPlate guidelines or the Half-Plate rule very difficult. That is, although they could control what they put on their plate, they were limited by what was put on the table in front of them.

## Discussion

There are three key conclusions and related implications for this study.

First, a simpler guidance system may be more effective than a complex one. When analyzing the people who rated the guidance system as easy to use versus easy to understand, it was found that the simpler the system was, the easier it was understood and the more correlated it was with selected healthier eating behaviors. Both of these simple visual icon-based guides were easier to understand and reduced eating-related questions than no system.

Second, even the most perfect dietary guidance system will not change behavior if the foods are not available. No dietary system will change behavior if only hamburgers and chips are available for lunch. What such a system can do is eventually encourage greater variety to be added that will balance out the meal.

Third, guidance systems may increase (for better or for worse) the consumption of any food they specifically mention and highlight, and they may even decrease the consumption of that food. Raising the awareness of foods that a person may not have otherwise eaten or eaten in significant quantities, such as dairy, meat, or starches, could unconsciously influence that person when he adheres to the systems specifically mentioned.

One powerful advantage of dietary guidance systems is that they may be one way to give a person more confidence in better knowing about how they can eat healthier [11]. Dietary guidance systems can help people move from mindless eating to more mindful eating [9], and the more simple the guidance system, the more confident a person feels when making healthy food choices. That may be one strong reason why some very simple-albeit controversial-systems, such as low carbohydrate diets, have been proven to be very popular for at least a brief period of time [12]. A simple rule as to what to eat - such as the MyPlate or the Half-Plate rule - can provide a bounded direction that encourages people to eat a wider variety of healthier foods.

Between these two dietary guidance systems, the Half-Plate rule, which unambiguously divides food into only two categories, was rated as easier to follow and understand than the MyPlate guidelines, which divide food into five different categories (5.41 versus 5.03). Moreover, it was generally found that the more understandable a dietary guidance system was to a person, the less meat, less dairy, and less dessert they consumed.

Although both dietary guidance systems left people with fewer questions on what they should eat, neither was particularly effective at dramatically changing how people ate. That is, they believed the dietary guidance systems were easier to understand, yet they did not always claim to eat better. A follow-up series of discussions with these individuals indicated that there were not always enough fruits and vegetables available on the table for them to eat in the way suggested by the guidelines. Using the Half-Plate rule can even be difficult if there is only one fruit or vegetable available. Perhaps Smart Homes could facilitate the availability of fruits and vegetables through technology or applications that help monitor food inventory or that provide recipe ideas that would help reduce this gap between understanding what to eat and doing it.

It was surprising to see, however, that the MyPlate guidelines led people to eat more dairy than they otherwise would. It might be that certain individuals who typically do not consume dairy in their normal meals were guided or reminded by the MyPlate guidelines to consider having dairy in their meals. This can be potentially advantageous in situations where people need to consume more calcium, such as elderly people, who are the focus of some of the many promising Smart Home projects. It can also be advantageous for children, people with very active or low iron levels, and people who require high-nutrition food portions for special hospital treatments. However, it may not be as advantageous to other groups of people who are already consuming too many calories and who may consume these products in the form of fattier dairy products (such as butter, whole milk, ice cream, and so on).

One of the strengths of this study was that the experiment was conducted in natural at-home dinners where participants could freely use dietary guidelines to decide what they ate. Yet, as an initial study in this area, there are several limitations to this study. First, because of the wide variation in eating conditions people experience, it would have been useful to conduct this study with a larger sample size that consisted of people of different ages and with different cooking capacities.

A second limitation of this study is that most people do not regularly keep a diet food diary. As a result, knowing that you will have to write down what you will be eating may alter the way a person eats a meal. Future studies can more carefully observe what people serve and how much they consume during meals, and they could also investigate how these variations might change across different meals. For instance, it might be expected that a guidance system might have less influence over breakfast or lunch than it does over dinner (where more food is available) across meal occasions. Furthermore, the variance in the demographics of our participants was not large in terms of education or economic background. Past research on MyPlate, for example, showed that the people who most quickly adapted to MyPlate were those who were the most educated and those who were the most attuned to their own dietary patterns [5].

## Conclusions

The Half-Plate rule and MyPlate were created as rough and ready tools that any person could use to eat better. They are simple guidelines to help people make real food decisions in real time (e.g., soup or salad; chicken or pasta), even if they do not have professional training in nutrition and dietetics. In this study, both the Half-Plate rule and MyPlate guidelines gave people more confidence in what to eat compared to using no system at all.

Dietary guidance systems are useful ways to encourage more mindful eating. Moreover, they can be easily modified to be used with apps and monitoring devices, or even in basic ways that are as simple as a reminder icon or graphic. The simpler and more flexible the advice, the more effective it will be. For instance, a half-plate rule that simply tells a person that half their plate should be fruit, veggies, and salad gives them the flexibility to eat healthier in a personal way that suits their tastes best.
